# A court ruled case on therapy‐induced false memories

**DOI:** 10.1111/1556-4029.15073

**Published:** 2022-06-02

**Authors:** Henry Otgaar, Antonietta Curci, Ivan Mangiulli, Fabiana Battista, Elisa Rizzotti, Giuseppe Sartori

**Affiliations:** ^1^ Faculty of Law and Criminology KU Leuven Leuven Belgium; ^2^ Faculty of Psychology and Neuroscience Maastricht University Maastricht The Netherlands; ^3^ Department of Education, Psychology, Communication University of Bari Aldo Moro Bari Italy; ^4^ Department of General Psychology University of Padua Padua Italy

**Keywords:** case, court, false memory, suggestion, therapy

## Abstract

We report on a unique Italian criminal case in which a court ruled that a therapist implanted false memories of abuse in a young girl. Using therapeutic excerpts, we show that the therapist used a multitude of problematic interventions that are all linked to false memory creation. Specifically, an analysis of the therapeutic excerpts showed that across many sessions, the therapist asked highly suggestive questions to the girl, implying that she was abused by her father. In addition, the girl underwent EMDR techniques that have been associated with memory undermining effects. Our analyses showed that although before treatment the girl did not have any recollection of being abused by her father, she gradually started to remember the abuse and identified the father as her abuser during the therapeutic sessions. Our case report clearly shows the danger of suggestive pressure in a therapeutic context causing patients to form false memories of abuse and supports the need to prevent the therapeutic practice of suggestive techniques.

**Highlights**

- This case report describes a unique case on therapy‐induced false memories.
- The case is unique as therapeutic records showed clear signs of suggestive treatment.
- The case is unique because the court ruled that the therapist implanted false memories.
- The case shows examples from research on how false memory are formed such as the effect of suggestion on false memory formation.

## 1 INTRODUCTION

People who are subjected to severe trauma, such as sexual abuse, frequently talk about their experiences with, for example, the police, child protection, or friends. During such interviews, questions are posed about what they can remember about the traumatic experience. On some occasions, such cases are brought into the courtroom. When this happens, an additional complexity is introduced as in the legal arena, it is vital to assess the accuracy of such testimonies of abuse. The assessment of this accuracy is imperative because legal decision‐making is often exclusively based on testimonies from victims, witnesses, or suspects [1].

A plethora of research has revealed that already from a young age (4–5 years old) people are able to accurately recount traumatic experiences [2, 3, 4]. However, research also suggests that memories can be tainted under suggestive conditions such as suggestive therapeutic interventions leading to so‐called false memories [5, 6, 7]. False memories are memories of details or events that were not experienced [6]. Such false memories can have devastating consequences as they can lead to false accusations and even end up in wrongful convictions. In this case report, we describe an Italian legal case in which the court ruled that a psychotherapist implanted false memories of sexual abuse in a young girl. Before we discuss this case more in depth, we will first present some important earlier legal cases regarding false memories and then explain how they fomented research into the area of false memory creation.

## 2 CASES ON FALSE MEMORIES

Empirical investigations into the formation of false memories commenced due to two intertwined developments that unfolded in the 1980s and 1990s. For example, in the 1990s, there was a bitter debate on the existence of unconscious repressed memory [8]. Repressed memory refers to the idea that because of the overwhelming nature of traumatic experiences, memories of these experiences are unconsciously blocked and not accessible for conscious introspection. One way that was heralded to exhume these unconscious repressed memories was therapy [9].

In the 1990s, several patients recovered memories of abuse after intense therapy. Some of them accused family members of abuse which even sometimes led to court cases. These cases spurred a heated debate about the authenticity of these recovered memories. Some clinicians claimed that certain mental and physical symptoms (e.g., sweating and anxiety) are indicative of buried memories of abuse and that therapeutic techniques such as hypnosis could be used to exhume these memories [10]. However, others—mostly memory researchers—argued that these interventions were suggestive possibly leading to the occurrence of false memories. This debate has been termed the memory wars, and there is mounting evidence that this debate on unconscious repressed memory rages on today [9, 11, 12].

Related to cases on recovered memories are so‐called daycare abuse cases. These are cases characterized by accusations of children concerning sexual abuse directed at one or a select group of suspects working at a daycare. A notable case is the McMartin preschool which took place in 1983 in Los Angeles [13]. In this case, hundreds of children reported to have been sexually abused by seven teachers. At the heart of this case, there were videotaped interviews with children that were led by a social service agency. The interviews showed extreme forms of suggestive pressure toward the children such as asking them about details that were never mentioned during any prior statements provided by the children (e.g., “Can you remember the naked pictures?”). Charges were eventually dropped because jurors concluded that the leading interviews contaminated children's testimonies.

In another American daycare abuse case, Kelly Michaels, a daycare worker, was convicted and sentenced to 47 years imprisonment in 1988 for sexually abusing 20 children [14]. Also, for this case, in 1993, the conviction was reversed because the argument was that children's statements were affected due to highly suggestive interviewing techniques. Such cases have not only occurred in Western countries. For example, in a recent case in 2016, at an elementary school in Jakarta (Indonesia), a teacher was sentenced to prison for sexual abuse toward children (see also https://coconuts.co/jakarta/news/mother‐alleged‐jis‐child‐abuse‐victim‐sues‐teachers‐5‐janitors‐idr1‐7‐trillion‐new‐civil‐lawsuit/). However, concerns were raised regarding the suggestive interviews with the children which likely led to false reports of abuse.

Even in Western countries, possible false memory cases are still lurking. For example, Shaw and colleagues (J. Shaw, personal communication, May 25, 2022) analyzed a large pool of cases in which possible false memories played a role. Specifically, they assessed a sample of 496 cases of the British False Memory Society, a foundation that provides support to people claiming to have been falsely accused of a crime (e.g., sexual abuse) potentially due to a false memory. The researchers found that in cases in which daughters accused their fathers, 84.31% (*n* = 153) of them underwent some form of therapy before the accusation. In the Netherlands, many cases involving potential false memories do not make their way to court. Instead, they are frequently evaluated by the Netherlands Expert Committee for Equivocal Sexual Abuse Allegations. This committee consists of different experts (e.g., investigative psychologists, cognitive psychologists, and clinical psychologists) who evaluate potential false memory cases and provide advice to the Public Prosecutor about whether an investigation should be continued in these cases. Recent data from this committee revealed that in the time period 2008–2020, 17% (*n* = 88) of the evaluated cases involved possible false recovered memories (N. Nierop, personal communication, May 25, 2022).

Collectively, cases such as the McMartin Preschool case show that, because of issues such as suggestive interviewing techniques, testimonies were deemed not reliable. Importantly, cases like these have stimulated research into paradigms and factors that lead to the formation of false memories.

## 3 FALSE MEMORY FORMATION

Various paradigms have been developed to create false memories. In general, these paradigms can elicit different types of false memories. For example, in the Deese‐Roediger/McDermott (DRM) paradigm [15, 16], participants learn lists of associatively related words (e.g., *tired, bed, dream, pillow, night, and slumber*). These words are connected to a non‐presented theme word called the critical lure (i.e., *sleep*). A common finding is that participants falsely recall/recognize the critical lure with rates sometimes as high as true memory rates [16]. False memories evoked by the DRM paradigm constitute spontaneous false memories as no external pressure is needed to foment them [1].

Alternatively, false memories that are elicited by the use of external factors frequently use some sort of suggestive pressure. For example, the misinformation paradigm is one of the most well‐known methods to induce suggestion‐based false memories [6]. In this paradigm, participants are presented with some stimuli (e.g., video of car crash). Following this, participants receive misinformation in the form of suggestive questions or narratives (e.g., an eyewitness testimony erroneously stating that an ambulance appeared while that was not the case). On a final memory test, some participants claim to remember having seen the misinformation during the encoding of the experience, an effect called the misinformation effect [17].

Another paradigm used to study suggestion‐based false memories is the false memory implantation paradigm [18, 19]. In this paradigm, participants are typically told that they experienced a false event (e.g., hot air balloon ride) in their childhood. During several suggestive interviews, about 30% of participants form false autobiographical memories for the event. Because these false memories concern autobiographical experiences, findings from this paradigm have been influential in discussions on therapy‐induced false memories of sexual abuse [20].

Of relevance for the current case report is research showing that Eye Movement Desensitization and Reprocessing (EMDR) has been shown to undermine the quality and quantity of memory [21]. EMDR is a popular therapeutic intervention in which patients need to retrieve their most distressing memory while they simultaneously have to horizontally follow the therapist’s index finger with their eyes. It has been shown that this eye movement procedure not only reduces the vividness and emotionality of autobiographical memories, but also has the potential to facilitate false memory creation [21].

Fully documented cases on false memory in legal settings are rare because of several reasons. First, in many cases, it is not completely certain what exactly happened during an event (i.e., there is no ground truth) meaning that caution should be exerted to call memories of abuse false. Second, it is challenging to write about cases as they can inadvertently jeopardize the anonymity of the involved parties. Third, although academics might have raised concerns about the possibility of false memory occurrence in certain legal proceedings, it is exceptional that courts themselves rule that memories are false memories. A case in point is a civil case in the Netherlands in which the court ruled that a therapist had to pay a financial compensation for implanting a false memory in a patient (see https://www.traumaversterking.nl/media21.html ). In the following, we will report on a unique Italian *criminal* case in which a therapist was convicted for implanting false memory of sexual abuse in a young girl perpetrated by her father.

## 4 AN ITALIAN CRIMINAL CASE ON A THERAPY‐INDUCED FALSE MEMORY

Here, we will report a court case in which evidence was available of false memory implantation. The case is unique in the sense that therapeutic sessions were recorded providing us with a unique insight in what happened during these sessions. The case attracted extensive media attention and most court documents are available over the internet. We made any effort in order to completely anonymize the case.

We will first sketch the general content of the case. Following this, we will highlight problematic therapeutic interventions of the case that fueled the production of false memories and will link these aspects with scientific research on false memories. To illustrate how these therapeutic interventions facilitated the formation of false memories, it is imperative to describe how the content of the memory changes throughout the therapeutic sessions. To this end, we will describe the victim's memory concerning the abuse before, during, and after the therapeutic sessions, and we will provide several representative extracts of therapeutic sessions. Finally, we will also provide quantitative analyses of the therapeutic sessions in order to show how the sessions unfolded and how complex they were for the patient.

The case concerned a psychologist and psychotherapist, Dr. X, who was charged for causing a girl to have serious psychological injuries under his therapeutic care. The criminal case is related to therapeutic sessions conducted in 2016. However, the story of this case already started in 2003 when the mother of Sara turned for the first time to the social services for economic and social support for a conflictual marital relation. Since then, the state of Sara's family had been under the lens of the social services. The name Sara was invented to assure anonymity, and therefore, we also do not use the name of the therapist.

In 2015, Sara was 15 years old, and her parents were divorced. In a meeting with the social workers, Sara's mother reported that she discovered that her daughter was sexually abused by her teenage boyfriend. The case was reported to the prosecutor and a criminal proceeding started against Sara's boyfriend. However, Sara did not want to be involved in that criminal proceeding and she was angry toward her mother for having revealed her confidence to the social workers. She was summoned by the social services and extensively questioned on the alleged abuse *before* she was heard in the court. Following these interviews, the girl reported that she felt a lot of shame because, in narrating her experiences to someone, and she finally realized how seriously she was affected by these experiences. At a later stage, social services informed Sara that, in childhood, she had been sexually abused by her father's friend, although the girl had no recollection of what happened.

In 2016, Sara was fully entrusted to the social services for a training project that included psychotherapeutic sessions with Dr. X, while the social workers charged for her care observed the progress of the clinical intervention through a mirror. When the therapeutic sessions started in February 2016, Sara reported some episodes, but Dr. X pushed her to state that these episodes were linked to sexual abuse carried out by a friend of her father when she was 5/6‐year‐olds, whose she had not had any memory *before* the psychotherapy. The sessions with Dr. X were videotaped, and this was critical for fully documenting the dynamics of the false memory implantation. At that time, Dr. X worked for a private center. This center was frequently asked to be involved in therapeutic care for problematic children by local services because of agreements with municipalities.

Sara's therapeutic treatment with Dr. X ran from February to October 2016. During 14 psychotherapeutic meetings, Dr. X addressed several times the theme of the alleged sexual abuses that Sara would have suffered on several occasions in her life by explaining the actual worries of the girl as a consequence of these abuses. More specifically, Sara was led to recall the abuse performed by her ex‐boyfriend at the age of 13–14, a recent aggression suffered by a classmate, and an abuse suffered by the father's friend in childhood (e.g., “Dr. X: *Is your father's friend who put his hands in a disturbing way?*”*;* see Table 1). During the sessions, the questions asked by Dr. X were often difficult to understand for Sara as they had a complex syntactic structure (see also below for the quantitative analyses on this). In addition, frequently, Dr. X's talks were long monologues aimed to persuade the girl that she was really sexually abused by her teenage boyfriend and the father's friend (e.g., “Dr. X: *By your father's friend. Eh… if you feel like it, ehm…. Sara, what do you remember of this abuse you suffered?*”; see Table S1). Because of the suggestive therapeutic interventions, she tended to yield to the psychotherapist's statements and assumptions, and frequently changed her answers to comply with his expectations (e.g., “Dr. X: *In some way he's doing a sexual act*. Sara: *Yes,*” see Table S3).

**TABLE 1:** Instances of suggestive questioning and acquiescent answers by Sara

Dr. X: Mh, older men, you say, eh? Maybe I'm dreaming up that… I don’t know it well, but it is possible that some older men have hurt you in the past.
Sara: (nods)
Dr. X: Eh, eh… so, to avoid such unpleasant risks you imagined taking the distance from everybody… didn’t you? Dr. X: …in a certain way, your father is also related with bad experiences of maltreatment and abuse?
Dr. X: He got his hands on you. So, it was a lack of respect, a serious lack of respect. I understand that your story… yes, indeed, it is not you…isn’t your life that sucks eh, eh, such men you met suck! You feel like you are mixing up the two things, eh?
Dr. X: Is your father's friend who put his hands in a disturbing way?
Sara: Yes.
Dr. X: On your body, eh?
Sara: Mh, mh.

*Note*: These excerpts are from the dialogues occurred during the psychotherapeutic sessions; therefore, side aspects (e.g., non‐verbal communication) and punctuation could be lost. In addition, dialogues were translated into English trying to be consistent with the original Italian version.

Furthermore, during the psychotherapy sessions, Sara underwent EMDR treatment by Dr. X. As long as the sessions progressed, in Sara's mind the figure of her father increasingly overlapped with that of his friend as the alleged abuser of her childhood (e.g., “Sara: *Ehm… I don't know why but… it happened to me quite often… ehm… that I confound my father's friend with my father*”; see Table S5). At a given point, Sara appeared more and more confused and uncertain whether the abuser was the father's friend or the father himself. Once the therapeutic sessions finished, Sara was invited by the social workers to continue to meet Dr. X twice a month. In October 2017, the Juvenile Court stripped Sara's father from his parental authority. After 2 years, in October 2019, Sara's mother and sister reported to the investigators that the girl had completely changed throughout the years, becoming irritable and aggressive, meeting untrustworthy friends, and also consuming drugs. Sara refused to meet her father and had a very conflicting relationship with her mother. She finally met her father in August 2019.

In November 2021, Dr. X was indicted of very serious injuries to Sara under his therapeutic care, abuse of office, and procedural fraud. The prosecutor had initially asked for a 6‐year sentence under the following criminal hypotheses. First, Dr. X was accused of having used strong suggestive questions affecting Sara's statements in a therapeutic setting to prove the occurrence of sexual abuse performed by her father which never occurred. Dr. X and his colleagues used non‐ethical and manipulative psychological techniques aimed at brainwashing children in order to induce victims to remember that they had been sexually abused by their parents. Among those techniques, emphasis was put on the use of highly suggestive interviews and alteration of ‘traumatic memories’ through EMDR practices.

During trial, it was reconstructed that children belonging to poor or problematic families were entered a psychotherapy program by Dr. X or one of his colleagues. Among the techniques adopted, the therapist disguised him/herself as a bad character of the most famous fairy tales and, dressed like this, acted as the father/mother of the child in a kind of cathartic approach. Children confronted with such characters were led to believe that their parents were dangerous, threatening, abusers, so that only facing them in such a symbolic act could free themselves from their discomfort.

Against the 6 years proposed by the prosecutor, the court sentenced Dr. X to 4 years in prison. He was also banned from public offices for a period of 5 years and cannot practice psychology and psychotherapy for 2 years. The court motivated the verdict in that, because of the “highly suggestive and inducive methods,” a false memory was implanted that Sara was abused by her father.

## 5 AN IN‐DEPTH ANALYSIS OF THE CASE

To more clearly delineate how Sara's memory changed through the therapeutic sessions, we will now describe some specific key examples before and during the therapeutic sessions by providing some excerpts of the therapeutic sessions supporting our claims.

### 5.1 Sara's memory for the abuse before the psychotherapy sessions

Before the therapeutic sessions with Dr. X, there was no evidence suggesting that Sara had any memory for being sexually abused by her father. Rather, she only claimed to have had sexual contacts with her teenage boyfriend.

### 5.2 Sara's memory for the abuse during the psychotherapy sessions

During the therapeutic sessions, Sara had a very passive attitude to respond to questions and frequently responded with a single word (e.g., “yes”). By contrast, Dr. X asked many suggestive and long questions. These suggestive questions were often related to abuse to which Sara responded in a mainly acquiescent way, modifying her answers in response to the psychotherapist's suggestions. The issue of abuse was indeed central to most of the sessions. In Table 1, we provide some examples of suggestive questions used by Dr. X.

A careful analysis of the dialogues between Dr. X and Sara clearly illustrates the changes in her memory over time. At first, Sara clearly explained that she had no recollection of the alleged abuse by the father's friend, and that this information was indeed acquired from the social services. More specifically, Sara stated that she had no memories for what happened surrounding the alleged abuse, except for an image of herself with a pink dress sitting on the sofa next to her father's friend. The image that Sara provided was fuzzy and not well placed in space and time. Moreover, Sara did not remember what the image in her memory referred to, and she did not know what specifically happened on that occasion. However, as long as the sessions with Dr. X proceeded, there was a progressive change in the girl's memory. The abuse suffered by the father's friend became clearer and referred as really experienced by herself (e.g., “Sara: *It turned to… not a picture anymore but like a short movie*”*;* see Table S4).

In the vague recollection provided by Sara in one of the first therapeutic sessions, there were only few details (e.g., the sofa, the pink dress, and father's friend). As the therapeutic program went on, Sara added more details (e.g., the face of the man and his hand) to the original memory and even included some central details in her accounts such as the hand of the father's friend touching her genital area.

During the therapeutic sessions with Dr. X, the abusive figure, initially identified as the friend of Sara's father, started to overlap with the father himself (e.g., “Sara: *Ehm… I can't… I can't understand why these two guys are always so similar…,*” see Table S5). At the end of the meetings, Sara finally recognized her father as the abuser. In this regard, the session of the 14th meeting is crucial. Using techniques from the treatment method called Eye Movement Desensitization and Reprocessing (EMDR), Dr. X led Sara to recover the memory of the abuse and in that moment the figure of the father's friend, until then considered the only responsible for the abuse suffered, took over to that of the girl's father. This is especially noteworthy as research has shown that certain EMDR techniques are linked to memory undermining effects and that EMDR therapists highly believe in the controversial concept of repressed memory [21]. In the Tables S1–S5, we provide some examples of different therapeutic sessions showing the alterations in Sara's memory.

### 5.3 Sara's memory for the abuse after the psychotherapy sessions

Suggestive questioning caused uncertainty in source attribution by Sara. As the excerpts in Tables S1–S5 show, after the therapy, the girl clearly stated that she cannot distinguish what happened with her father from what happened with her father's friend (e.g., *“Sara: I don't know why I see too much similarity with my father, so I don't know”*; see Table S5).

### 5.4 Additional analysis of the therapeutic sessions

Apart from the observation that Dr. X used highly suggestive prompts during his interventions, Dr. X also asked detailed questions containing many words which might have been an additional load to Sara's comprehension of the therapy. For example, on average, Dr. X reported a total of 3291 words, while the patient, Sara, used a total of 1219 words.

Furthermore, on average, Dr. X used 21.11 words per sentence, while Sara 12.28. These observations clearly reveal that there was a strong imbalance concerning the amount of information shared between Dr. X and Sara, with Dr. X contributing the most to the therapy sessions (For a detailed quantitative analysis, follow this link: https://osf.io/p39by/).

## 6 IMPLICATIONS FOR POLICY, LAW, AND SCIENCE

The present case report leads to several implications for policy, the legal arena, and the scientific field. First of all, our article further underlines the need to keep investigating how therapeutic interventions might exert ramifications for false memory propensity. That is, research has revealed that certain types of therapies such as hypnosis might be suggestive, thereby possibly accelerating false memory formation (e.g., [10]). However, less attention has been devoted to the issue whether certain empirically based treatments contain elements that might prompt the risk for false memory formation. For example, eye movements as used EMDR have been related to changes in memory quality and quantity [21]. Other widely used therapies also contain techniques that involve the retrieval of memories [22, 23]. For example, imagery rescripting is a therapeutic intervention in which patients have to rescript or change their traumatic memories toward a more positive one [24]. Research still has to commence into examining how such techniques might have the potential to instill false memories.

Second, the issue on how false memories can be evoked using therapeutic interventions comes close to the question whether therapies have potential negative side effects [25]. That is, although much empirical research has been focused on the positive aspects of treatments, limited research exists on whether treatments might be harmful. Especially interesting in this regard is research concerning the relation between therapy and any known side effects. For example, Dandachi‐Fitzgerald and colleagues [26] surveyed (former) patients about any negative and positive side effects due to treatment. They found that the patients reported a median of 6 negative and 13 positive therapy experiences. Of interest for the current case study is that 27% (*n* = 54) of the (former) patients indicated that negative memories were recovered during therapy that were unknown before therapy. Growing awareness that therapies might cause harm can be beneficial for patients in deciding which therapeutic orientation to choose.

Third, in terms of policy implications, our case report evidently addresses a scientist–practitioner gap [27]. Specifically, in this case, it is obvious that the therapist was likely unaware of the perilous effects of suggestive questions. This observation mirrors recent research on whether therapists might use suggestive techniques during treatment. For example, Patihis and Pendergrast [28] asked 2326 US citizens whether they ever had therapy and if so, whether their therapist ever discussed the existence of repressed memories. The authors found that 9% of the total sample reported that this had happened during their treatment (see also [29]). Thus, this case stresses the need of appropriate training and educational programs for therapists on the science of memory. Note that these recommendations have been made by other scholars as well (e.g., [12]). Interestingly, recent research shows that such education can make people more critical toward controversial aspects of memory (e.g., repressed memory). For example, Sauerland and Otgaar [30] recently demonstrated that providing students with education on the science of memory (i.e., series of lectures) led them to be more critical toward believing in repressed memory as compared to before receiving such education. Although no work has been conducted with therapists, Sauerland and Otgaar's findings are promising as they imply that education on the science of memory should become an integral part of curricula of mental health professions.

Finally, regarding the legal implications, our case report shows that in instances concerning the reliability of testimonies of abuse, memory experts might play an essential role in educating the court about how suggestive therapeutic interventions might catalyze the formation of false memories. This is especially relevant as research shows that judges, because of their limited knowledge in the area of memory, are often ill‐equipped to infer whether statements refer to an authentic or fabricated experience (e.g., [31]). Our argument is that when legal professionals (e.g., judges, lawyers, and prosecutors) have concerns or questions pertaining to the testimonial accuracy of alleged victims, witnesses, and suspects, they should consult memory experts [32]. These experts might provide the courts with general or case‐relevant information about the reliability of memory.

## 7 CONCLUDING REMARKS

Although several concerns have been raised about the possibility that some therapists might suggestively induce false memories in patients (e.g., [33]), well‐documented case studies of such therapy‐induced false memory creation are rare (see also [34]). The current report provides a unique glimpse into an Italian case in which a therapist was convicted for causing psychological damage because of using highly suggestive methods. These methods made the teenaged patient to falsely remember being abused by her father. She then went to therapy because of alleged traumatic experiences in her childhood. She had no recollection of any abuse perpetrated by her father before she entered therapy. However, the case report showed that during the therapeutic sessions, the therapist suggested numerous times that her father did abuse the girl during her childhood. The sessions clearly show that although the girl is reluctant about this abuse scenario, she gradually began to accept this suggestion, thereby eventually forming a false memory for being sexually abused by her father.

The therapeutic sessions contained several problematic elements that might have catalyzed the creation of a false memory. First and foremost, during the sessions, many suggestive questions and statements were posed (e.g., “Dr. X: *In some way he's doing a sexual act,” “it is possible that some older men have hurt you in the past*”). A wealth of experimental research has shown that such suggestions can lead to source monitoring failures, thereby engendering false memories [6, 17] Second, in the sessions, there were signs that the girl was explicitly encouraged to imagine certain experiences (e.g., “Dr. X: *[…] to avoid such unpleasant risks you imagined taking the distance from everybody”*). This is noteworthy as research has revealed that imagining fictitious events can lead to imagination inflation in which people become confident and even form false memories that these events took place [35, 36]. Third, the therapist made the use of EMDR techniques during treatment. This is interesting in light of recent studies showing that eye movements as applied in EMDR can affect the memory quality and quantity of experiences [21]. For example, research has shown that eye movements can lead to the production of spontaneous false memories [37] (but see also [38]). Finally, our quantitative analyses show that there was a strong imbalance in terms of the words spoken by the girl and the therapist. Specifically, we found that the therapist used significantly more words which were also grammatically difficult than the girl which might have contributed to the girl forming a false memory of abuse. That is, because of the many suggestive questions posed in a difficult manner by an authority (i.e., therapist), this might have made Sara to go along with these suggestions. One reason is that such difficult questions might have hampered efficient communication and confusion for Sara, which might have amplified her willingness to go along with the suggestion [39].

From a forensic point of view, this case shows that inappropriate interview techniques adopted in therapeutic settings can negatively affect a patient's mental health. That is, Sara's mental health was profoundly damaged by the therapeutic interventions carried out by Dr. X and her life was drastically changed along with her family relations. This is the first criminal case, to our knowledge, where a court ruled out that serious injuries were done on victims' memories by use of therapy.

A final point that deserves comment is that therapies are frequently person‐centered [40]. The consequence of this is that therapists focus on patients' subjective experience and that it does not matter whether these experiences are authentic or not. In our opinion, this is problematic. Our reasoning is that in the therapeutic context, it is important to be cognizant of the situation that patients might sometimes have memories for traumatic events that were not experienced. Such awareness might minimize the chance that suggestive interventions are set in motion which might ultimately lead to false memories of sexual abuse.

## Supporting information


Tables S1‐S5
