# Supplementary material for: A court ruled case on therapy‐induced false memories
Source: J Forensic Sci. 2022 Jun 2;67(5):2122–9. doi: 10.1111/1556-4029.15073 (PMC9544012; doi:10.1111/1556-4029.15073)
Supplement: Supplementary file 1 — Tables S1‐S5 [file JFO-67-2122-s001.docx]

**Appendix**

TABLE S1 Therapeutic sessions between Dr. X and Sara

| Dr. X: Then, your father to what is still related to?  Sara: To the first abuse I suffered.  Dr. X: By your father’s friend?  Sara: Yes.  Dr. X: By your father’s friend. Eh... if you feel like it, ehm.... Sara, what do you remember of this abuse you suffered?  Sara: Mh... I only have one picture fixed in my mind, that is this man did hurt me, I knew it from Kathy...  […]  Dr. X: In this idea... ehm... (sighs) about the idea of this friend of your father, what did Kathy tell you?  Sara: That... ehm... if I am not mistaken, she told me that when I was young, I had already been there for something I told to my aunt, about what the friend of my father did to me.  […]  Dr. X: Ah, ah. You... ehm... You don’t remember anymore but Kathy told you that when you were a child you said….  Sara: Yes.  Dr. X: That your father’s friend did something to you.  Sara: Mh, mh.  Dr. X: Do you remember what Kathy told you that you said?  Sara: I don’t remember.  Dr. X: *You don’t remember.*  Sara: But last time my mother told me about this, as I first told the story to my aunt, then my aunt referred it to my mom, she was worried, and we went to them, my father said that... mh... I mean… that I made it all up.  Dr. X: But don’t you remember anything of this? It’s just Kathy’s story or is there anything from you too? Something you remember even personally I would say…  Sara: Mh.... I don’t know.  Sara: But anyway, when I was used to go to this person’s place, I didn’t feel comfortable, that is I have this picture of me on the sofa next to my father’s friend with a dress, if I am not wrong; I remember that.  Dr. X: A dress that...did this dress have any peculiarities?  Sara: Eh... pink. |
| --- |

TABLE S2

| Dr. X: […] What do you remember, what images do you have?  Sara: That I was in his place, of this person, he’s close to me, I think.  Dr. X: Ah ah.  Sara: …he puts his hand here nearby (she shows the part of her body from her stomach to the genitals) but not... that is I’m not... I don’t know how I’m seeing that, I’m not the person who I’m watching.  Dr. X: As if you are seeing the scene from the outside. |
| --- |

TABLE S3

| Dr. X: What do you see in this image?  Sara: Ehm...he’s close to me.  Dr. X: Ah ah.  Sara: Ehm... that he’s doing... mh (Sara covers her face with her hands) ehm... I don’t know how to say it.  Dr. X: In some way he’s doing a sexual act.  Sara: Yes.  Dr. X: He’s doing a sexual act. On him or on you?  Sara: On me.  Dr. X: On you. He’s doing a sexual act on you.  Sara: Well, he’s not doing anything yet, but he’s about to do something.  Dr. X: About to do. This sexual act is getting closer.  Sara: Yes.  Dr. X: In this scene, do you see his genitals, for instance, or not?  Sara: Ehm...  Dr. X: No, you don’t see them. Do you see his face, his hands, which parts of his body are evident?  Sara: Ehm... not his face. I see from here to... (she shows a part of her body from her neck to the pelvis).  Dr. X: The picture is cropped. The picture is cropped.  Sara: Yes.  Dr. X: You can’t see his face, can you see his body down to…?  Sara: More or less down to here (she indicates her knees). He’s sitting, so it can be seen... (again she indicates her knees with her hands).  Dr. X: Sitting, eh, how does his body move, how do his hands move? In this scene how are his hands? How is his body? Is he sitting then? Is there anything that’s moving, from what you’re saying to me?  Sara: His hand.  Dr. X: His hand. His right hand, his left hand? I think you remember, that’s not very important, by the way...  Sara: His right hand... (she covers her eyes)... no, his right hand... mh, I think it is his right one but...  Dr. X: You do believe it’s his right hand.  Sara: Yes.  Dr. X: Is a hand moving?  Sara: Yes.  Dr. X: It’s moving, could it be his hand?  Sara: I see it here (She indicates her vaginal area with her hand), in this way.  Dr. X: A hand already launched, directed to your body.  Sara: Yes.  Dr. X: In this scene do you see your body too?  Sara: Yes.  Dr. X: How is your little girl’s body?  Sara: Ehm... I’m so young.  Dr. X: Really young.  Sara: Ehm... with an innocent air.  Dr. X: Ah ah, ah ah, consistent with your age, right? Was this recoded in your memory picture, eh? Can you see your little dress too?  Sara: Ehm... yes.  Dr. X: What dress is it?  Sara: If I correctly remember, it was pink.  Dr. X: Pink?  Sara: Yes.  Dr. X: Is it pink?  Sara: Yes, I was dressed in pink.  Dr. X: Is the hand of this man touching your little dress, is it close by?  Sara: Ehm... really close.  Dr. X: Really close, really close, eh. Before precisely describing this picture, I first ask you if there were any details, if there were still any details in this picture?  Sara: Ehm... well, I see the wall behind the sofa.  Dr. X: Dang! So many things!  Sara: The sofa and nothing else. |
| --- |

TABLE S4

| Sara: Ehm...I can’t see his face but he invited me to the sofa, he did like this (a gesture with her hand) "Sit down" and... then stop, I was at that point.  Dr. X: Very well done, very good, with no effort, what appears, what do you notice, okay? Let’s go ahead (he moves his fingers to go on).  Sara: Ehm... I asked me if I’d love him.  Sara: It turned to... not a picture anymore but like a short movie.  Sara: Yes, well... but there’s something... that is I see... more or less I see his face, I always see a kind of mix between my... my father and his friend. |
| --- |

TABLE S5

| Sara: Ehm... I don’t know why but... it happened to me quite often... ehm... that I confound my father’s friend with my father.  Dr. X: Ah ah, ah ah. |
| --- |
| Sara: But I am not sure of this, I don’t know why sometimes there’s this switch. |
| Dr. X: Ah ah, this overlapping, this switch, yes. |
| Sara: Yes, so... I don’t know. |
| Dr. X: But did you notice this overlapping, eh? You’re telling me about this overlapping you made oftentimes. So, I’d say to remain on this overlapping, let’s start from this overlapping between this friend and your father, let’s see what you notice. |
| Sara: Ehm... I can’t... I can’t understand why these two guys are always so similar... |
| Dr. X: Mh mh. |
| Sara: That’s it, I’d like to see his face but I can’t, I don’t know why, I can only see... |
| Dr. X: Ah ah, in the picture there isn’t any face, you still can’t see it, ah ah. If you think of that picture, can you still see the face? Is such an image changing somehow? |
|  |
| Sara: Ehm... I can remember I would say this bogeyman... ehm... that is he has a weird grimace, he has that face... ehm...it’s always switching with that of my father. |
| Dr. X: Ah ah. |
| Sara: Well, it’s more the face of my father’s friend, but I don’t know why I see too much similarity with my father, so I don’t know. |

**Note:** These are excerpts from the dialogues occurred during the psychotherapeutic sessions, therefore side aspects (e.g., non-verbal communication) and punctuation could be lost. In addition, dialogues were translated into English trying to be consistent with the original Italian version.
